# Carbon dioxide capture from air leading to bis­[*N*-(5-methyl-1*H*-pyrazol-3-yl-κ*N* ^2^)carbamato-κ*O*]copper(II) tetra­hydrate

**DOI:** 10.1107/S2056989023008575

**Published:** 2023-10-05

**Authors:** Valerii Y. Sirenko, Iryna S. Kuzevanova, Oleksandr S. Vynohradov, Dina D. Naumova, Sergiu Shova

**Affiliations:** a Department of Chemistry, Taras Shevchenko National University of Kyiv, Volodymyrska str. 64/13, 01601 Kyiv, Ukraine; b Department of General and Inorganic Chemistry, National Technical University of Ukraine "Igor Sikorsky Kyiv Polytechnic Institute", Peremogy Pr. 37, 03056 Kyiv, Ukraine; c Innovation Development Center ABN, Pirogov str. 2/37, 01030 Kyiv, Ukraine; d Department of Inorganic polymers, "Petru Poni" Institute of Macromolecular Chemistry, Aleea Gr. Ghica, Voda 41A, 700487 Iasi, Romania; University of Neuchâtel, Switzerland

**Keywords:** 5-methyl-3-pyrazolamine, copper(II) acetate, di­ethano­lamine, Hirshfeld surface analysis, crystal structure, copper(II) complexes

## Abstract

A mononuclear square-planar Cu^II^ complex of (5-methyl-1*H*-pyrazol-3-yl)carbamate, [Cu(C_5_H_6_N_3_O_2_)_2_]·4H_2_O, was synthesized using a one-pot reaction from 5-methyl-3-pyrazolamine and copper(II) acetate in water under ambient conditions. The adsorption of carbon dioxide from air was facilitated by the addition of di­ethano­lamine to the reaction mixture. While di­ethano­lamine is not a component of the final product, it plays a pivotal role in the reaction by creating an alkaline environment, thereby enabling the adsorption of atmos­pheric carbon dioxide. The central copper(II) atom is in an (N_2_O_2_) square-planar coordination environment formed by two N atoms and two O atoms of two equivalent (5-methyl-1*H*-pyrazol-3-yl)carbamate ligands. Additionally, there are co-crystallized water mol­ecules within the crystal structure of this compound. These co-crystallized water mol­ecules are linked to the Cu^II^ mononuclear complex by O—H⋯O hydrogen bonds. According to Hirshfeld surface analysis, the most frequently observed weak inter­molecular inter­actions are H⋯O/O⋯H (33.6%), H⋯C/C⋯H (11.3%) and H⋯N/N⋯H (9.0%) contacts.

## Abstract 2

A mononuclear square-planar Cu^II^ complex was synthesized by reacting 5-methyl-3-pyrazolamine and copper(II) acetate in water under ambient conditions. Di­ethano­lamine was added to facilitate carbon dioxide adsorption, creating an alkaline environment. Structural analysis revealed that the complex crystallizes in the *P*2_1_/*c* space group of the monoclinic crystal system, with the central copper(II) atom in a square-planar coordination environment N_2_O_2_. Co-crystallized water mol­ecules are present, forming O—H⋯O hydrogen bonds with the Cu^II^ mononuclear complex. Hirshfeld surface analysis highlighted the importance of various inter­actions, including H⋯O/O⋯H, H⋯C/C⋯H, and H⋯N/N⋯H, in providing crystal structure packing.

## 1. Chemical context

Currently, global warming stands out as the most significant environmental concern, leading to climate change and giving rise to a range of effects, including elevated sea levels, prolonged droughts, intensified hurricanes, and a surge in extreme weather occurrences (Ochedi *et al.*, 2021 ▸). The primary cause of global warming in recent decades can be attributed to the heightened levels of greenhouse gases in the atmosphere, with particular emphasis on the concentration of CO_2_ (Aghaie *et al.*, 2018 ▸). Power plants, comprising more than 40% of CO_2_ emissions, with coal-fired facilities accounting for 73% of fossil fuel-based power plant emissions (Cannone *et al.*, 2021 ▸; Mikkelsen *et al.*, 2010 ▸), are a significant contributor to the carbon footprint. Given the widespread use of fossil fuels, particularly coal, there is a strong need to develop effective methods for capturing and mitigating CO_2_ emissions from power plant flue gases, to help stabilize the atmospheric CO_2_ level (Wang *et al.*, 2017 ▸).

Various technologies, including adsorption (Milner *et al.*, 2017 ▸), absorption (Conway *et al.*, 2013 ▸), membrane separations (Sreedhar *et al.*, 2017 ▸), cryogenic distillation (Song *et al.*, 2019 ▸), and chemical looping (Kronberger *et al.*, 2004 ▸), are currently under research and development for capturing CO_2_ from flue-gas streams. One potential strategy for reducing carbon emissions in the future involves the utilization of carbon capture and sequestration (CCS) materials.

The process of CCS entails the specific separation and subsequent storage of CO_2_ taken from exhaust gas mixtures, which predominantly consist of N_2_, CO_2_, H_2_O, and O_2_, preventing their release into the atmosphere. Following this, the collected CO_2_ is transported for either utilization or long-term storage. Amine scrubbing-based chemical capture methods have garnered significant focus and inter­est (Tang *et al.*, 2005 ▸; Mani *et al.*, 2006 ▸).

One of the methods for reducing carbon dioxide levels in the environment involves capturing it through the formation of carbamates (Conway *et al.*, 2011 ▸; McCann *et al.*, 2009 ▸; Zhang *et al.*, 2017 ▸). Besides, carbamates can be used as catalysts or useful inter­mediates in the synthesis of other, more-valuable chemicals (Dell’Amico *et al.*, 2003 ▸). Given the necessity of capturing CO_2_ to address broader societal needs, in this article we report the synthesis, crystal structure and Hirshfeld surface analysis of a new mononuclear copper(II) complex with (5-methyl-1*H*-pyrazol-3-yl)carbamic acid – [Cu(5-MeHpzCarb)_2_]·4H_2_O. 
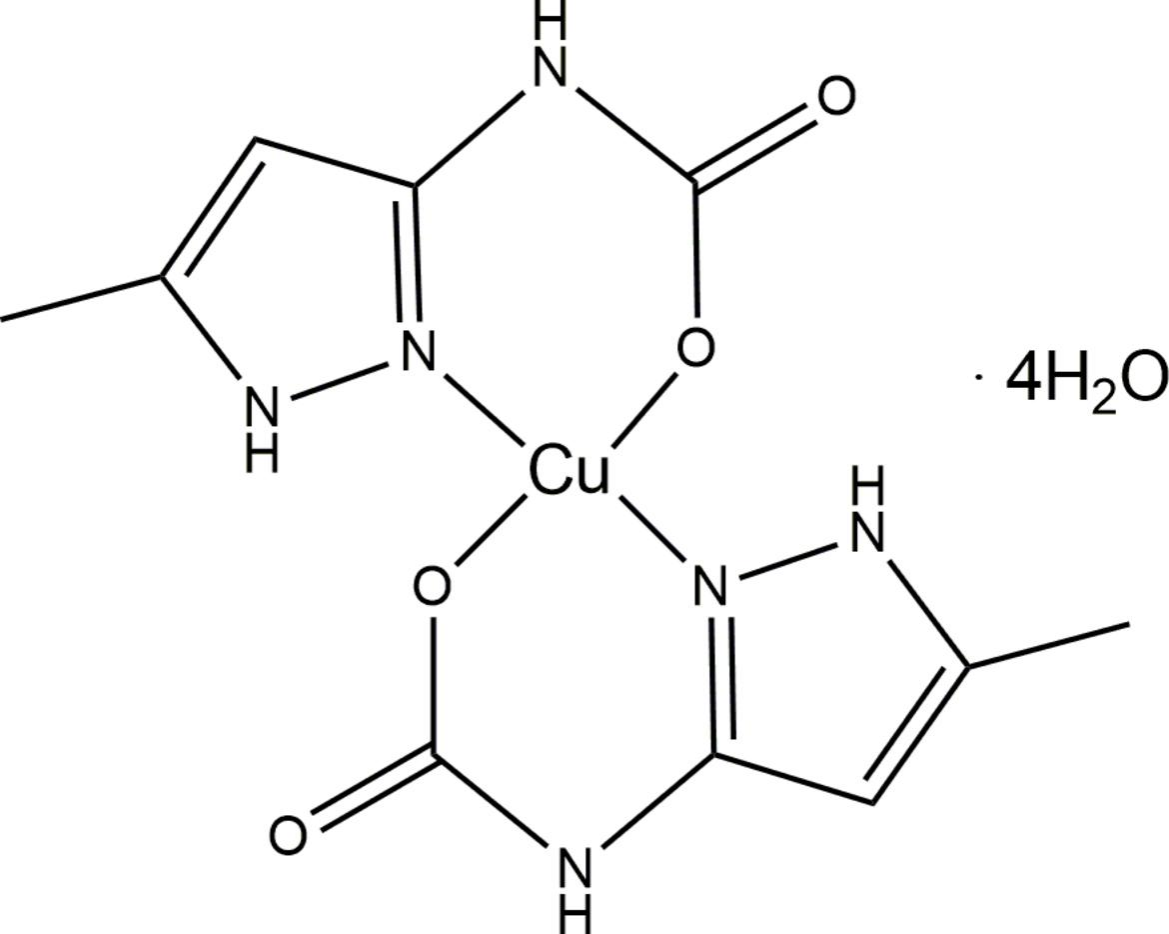


## 2. Structural commentary

**Figure 1:**
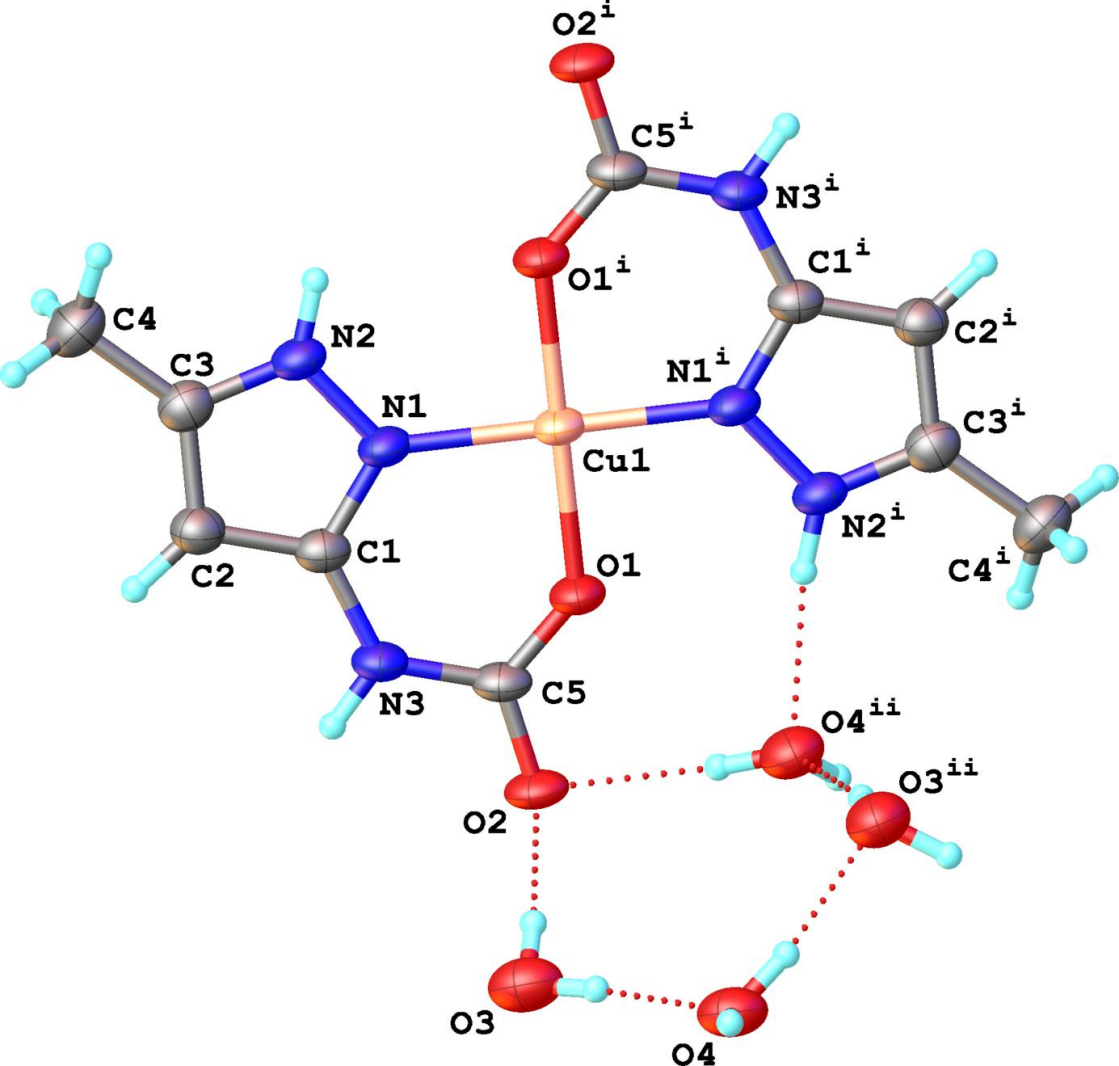
Representation of the [Cu(5-MeHpzCarb)_2_] complex and co-crystallized water mol­ecules, showing the atom-labelling scheme and displacement ellipsoids drawn at the 50% probability level. H atoms are shown as small spheres of arbitrary radii. Symmetry codes: (i) −*x*, −*y*, −*z*; (ii) 1 − *x*, − 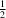 + *y*, 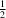 − *z.*

The title compound crystallizes in the monoclinic space group *P*2_1_/*c*, and has a crystal structure built upon neutral mononuclear [Cu(5-MeHpzCarb)_2_] units (Fig. 1 ▸). Co-crystallized water mol­ecules are present in a 1:4 ratio to the complex as inter­stitial mol­ecules. The asymmetric unit includes one copper site (SOF is 0.5, Wyckoff position 2a), one (5-methyl-1*H*-pyrazol-3-yl)carbamate ligand and two co-crystallized water mol­ecules.

The Cu^II^ ion displays a square-planar coordination environment (N_2_O_2_) formed by two nitro­gen atoms of pyrazole rings and two oxygen atoms of carboxyl­ate group of (5-methyl-1*H*-pyrazol-3-yl)carbamate ligands. The Cu1—N1 distances are 1.931 (2) Å while the Cu1–O1 distances are shorter and account to 1.9140 (17) Å. The O1–Cu1–O1^i^ and N1—Cu1—N1^i^ bond angles are 180°, which is typical for a square-planar arrangement (Fig. 1 ▸). At the same time, the N1—Cu1—O1^i^ and N1—Cu1—O1 bond angles slightly deviate from the ideal value of 90°, which is the result of the formation of the six-membered chelate rings. Selected bond lengths and bond angles are given in Table 1 ▸. The Cu1 atom lies within the plane defined by N1—O1—N1^i^—O1^i^. Additionally, the Cu atom lies within the planes of the aromatic rings, whereas O1 and O1^i^ are slightly above the plane, with an O1(O1^i^)-to-plane distance of 0.182 (3) Å.

In the crystal structure, monomeric [Cu(5-MeHpzCarb)_2_] units form layers with Cu1 centres lying in the *ab* plane. The plane-normal-to-plane-normal angle between the horizontal N1—O1—N1^i^—O1^i^ planes of two adjacent layers is 74.762 (2)°.

## 3. Supra­molecular features

**Figure 2:**
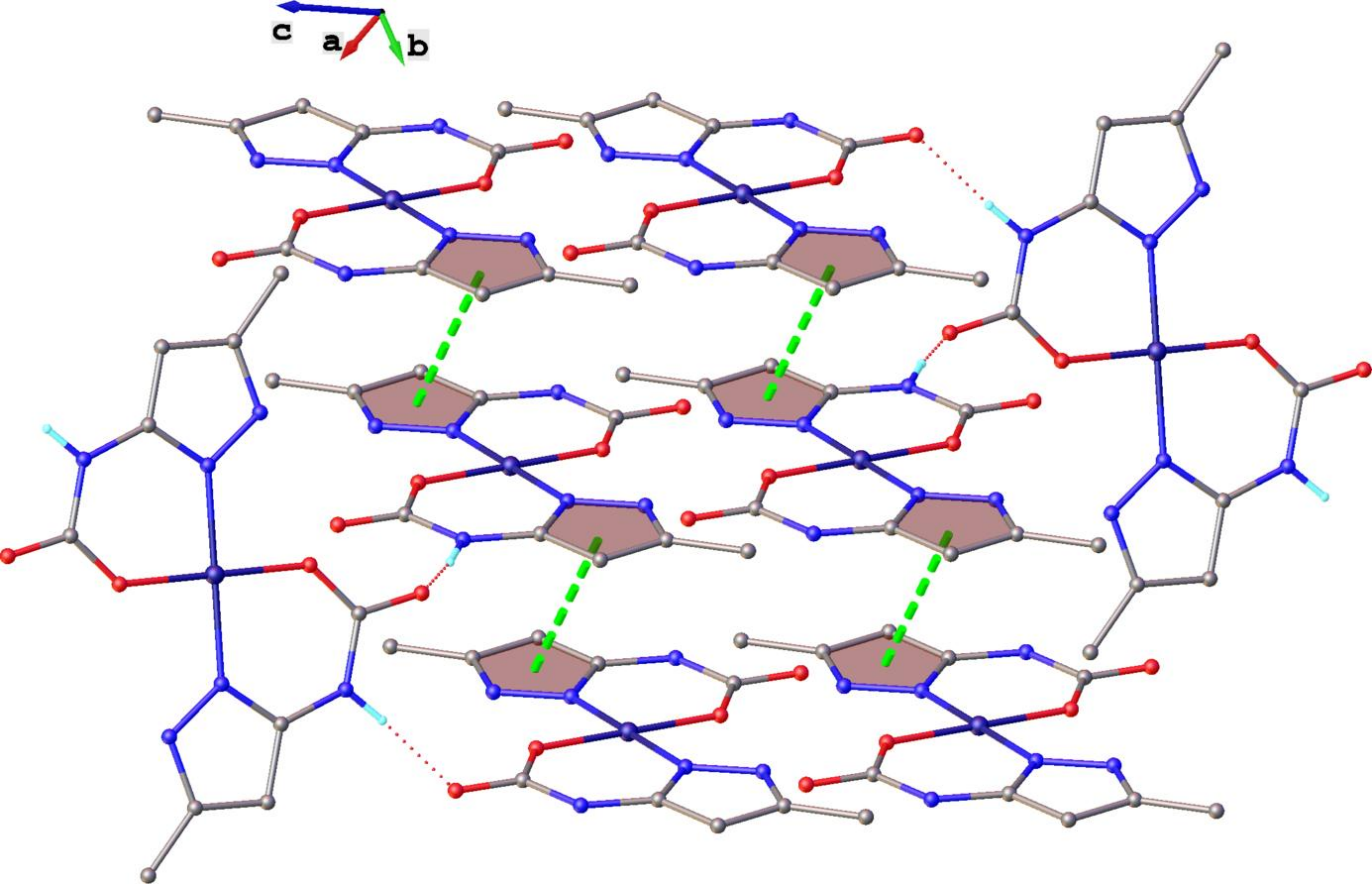
Partial crystal packing of [Cu(5-MeHpzCarb)_2_]·4H_2_O showing inter­molecular π–π and N—H⋯O contacts as green and red dashed lines, respectively.

All the components of the structure are associated *via* inter­molecular O—H⋯O and N—H⋯O hydrogen bonds, as well as weak C—H⋯O contacts (Figs. 2 ▸, 3 ▸). π–π contacts are also observed between neutral [Cu(5-MeHpzCarb)_2_] mol­ecular complexes (Fig. 2 ▸). The co-crystallized water mol­ecules are inter­leaved with the supra­molecular layers of the neutral [Cu(5-MeHpzCarb)_2_] complexes along the *c*-axis. The O4 water mol­ecule participates in four hydrogen bonds, two where it acts as a donor (O4—H4*E*⋯O2^ii^ and O4—H4*D*⋯O3^i^, see Table 2 ▸ for details), and two as acceptor (O3—H3*B*⋯O4 and N2—H2⋯O4^iii^, see Table 2 ▸ for details). At the same time, the O3 water mol­ecule participates in three hydrogen bonds, two where it acts as a donor (O3—H3*A*⋯O2 and O3—H3*B*⋯O4, see Table 2 ▸ for details) and one as acceptor (O4—H4*D*⋯O3^i^, see Table 2 ▸ for details). In addition, the O3 water mol­ecule participates in a weak C2—H2*A*⋯O3^iv^ contact with a C2⋯O3 distance of 3.340 (4) Å. According to this, the co-crystallized water mol­ecules play an important role in providing cohesion between the neutral [Cu(5-MeHpzCarb)_2_] mol­ecular complexes. Geometric parameters for inter­molecular hydrogen bonds are given in Table 2 ▸.

**Figure 3:**
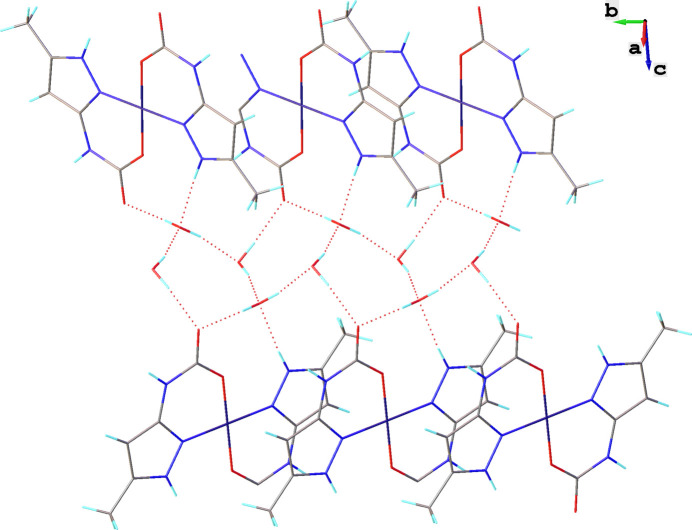
Partial crystal packing of [Cu(5-MeHpzCarb)_2_]·4H_2_O showing the five-membered supra­molecular ring formed by four water mol­ecules and the carboxyl group of the (5-methyl-1*H*-pyrazol-3-yl)carbamate ligand.

Inter­estingly, four water mol­ecules and the carboxyl group form a five-membered supra­molecular ring (Fig. 3 ▸). In addition, π–π inter­actions are observed between the [Cu(5-MeHpzCarb)_2_] neutral complexes. The plane-to-plane distance for these π–π contacts is 3.324 (3) Å with the plane-to-plane shift being 1.498 (5) Å. It is also worth noting very weak C—H⋯π contacts between two contiguous [Cu(5-MeHpzCarb)_2_] units with a carbon-atom-to-plane distance of 3.586 (4) Å.

## 4. Hirshfeld surface analysis

**Figure 4:**
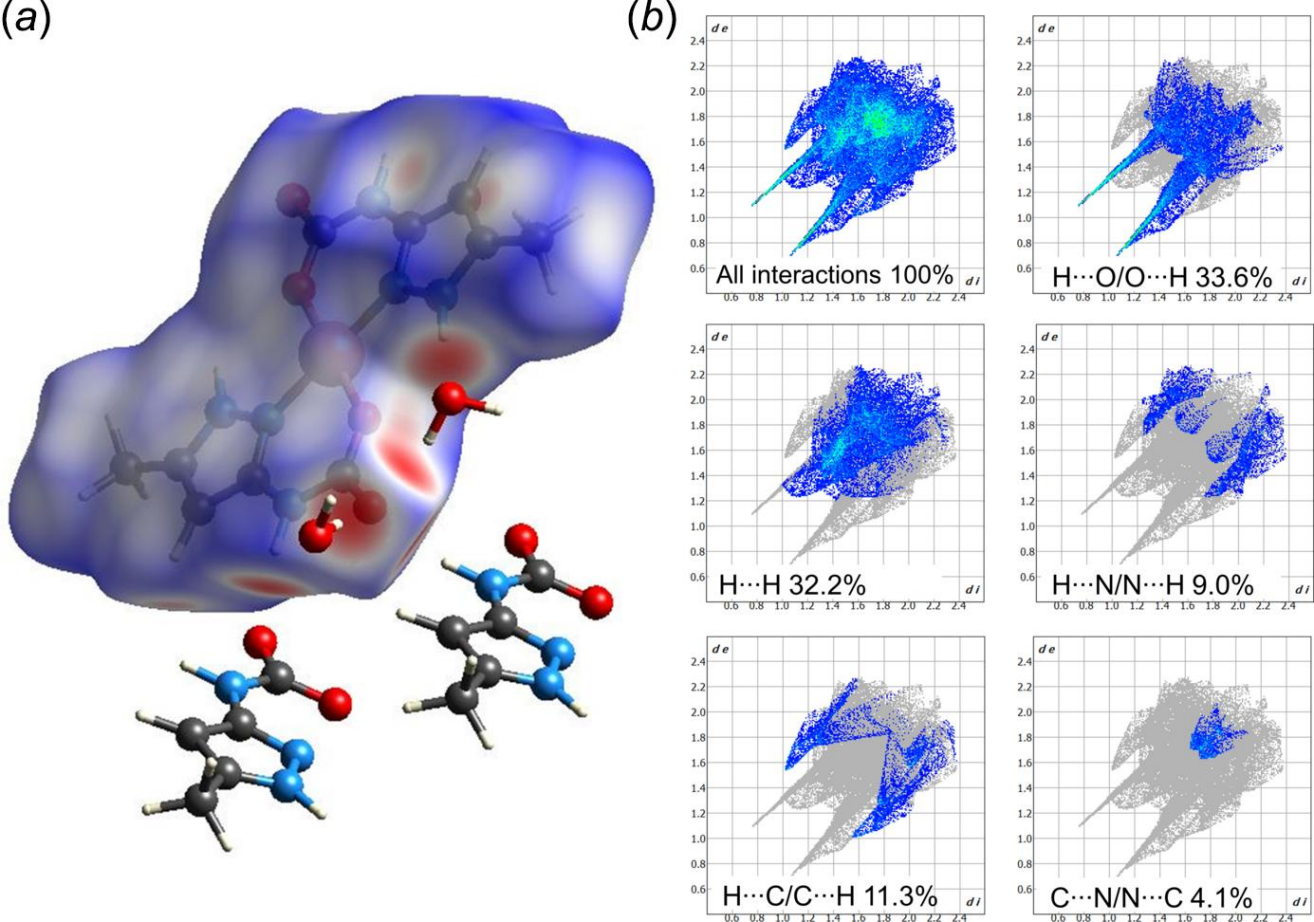
(*a*) Hirshfeld surface representations with the function *d*_norm_ plotted onto the surface for the different inter­actions; (*b*) two-dimensional fingerprint plots, showing the contributions of different types of inter­actions.

The Hirshfeld surface analysis was performed and the associated two-dimensional fingerprint plots were generated using *Crystal Explorer 21.5* software (Spackman *et al.*, 2021 ▸), with standard resolution of the three-dimensional *d*_norm_ surfaces plotted over a fixed colour scale of −0.6468 (red) to 1.1041 (blue) a.u. There are eight red spots on the *d*_norm_ surface (Fig. 4 ▸ *a*). Visualizations were performed using a red–white–blue colour scheme, where red highlights shorter contacts, white is used for contacts around vdW separation, and blue depicts longer contacts. The red spots on the 3D *d*_norm_ Hirshfeld surfaces indicate the direction and strength of the inter­molecular *E*—H⋯O hydrogen bonds (where *E* = N, O), as well as weak C—H⋯O and C—H⋯π contacts. The overall two-dimensional fingerprint plots for the selected inter­actions are shown in Fig. 4 ▸ *b*.

The most significant contributions to the overall crystal packing are from H⋯H (32.2%), H⋯O/O⋯H (33.6%), H⋯C/C⋯H (11.3%), H⋯N/N⋯H (9.0%) and C⋯N/N⋯C (4.1%) inter­actions. The H⋯O/O⋯H contacts form a pair of spikes on the sides of the corresponding two-dimensional plot, which are indicative of strong inter­molecular inter­actions between atoms. At the same time, the H⋯N/N⋯H and H⋯C/C⋯H contacts form less pronounced spikes, indicating that these inter­actions are weaker.

## 5. Database survey

A search of the Cambridge Structure Database (CSD version 5.44, last update June 2023; Groom *et al.*, 2016 ▸) revealed that the structure has never been published before. 51 structures for the Cu(pyrazole)_2_(CO_2_)_2_ moiety [four-coordinated copper atom with an N_2_O_2_ coordination environment] were found. Most similar to the title compound, complexes forming a four-coordinated N_2_O_2_ coordination environment, are *trans*-bis­(3,5-di­methyl­pyrazole)­bis­(pivalato)copper(II) (DEFSAJ; Zhou *et al.*, 2006 ▸), bis­(1*H*-indazole-3-carboxyl­ato)copper(II) (ETOVUH; Qin *et al.*, 2017 ▸), *trans*-bis­(4-nitro­benzoato-*O*)bis­(3,5-di­methyl­pyrazole-*N*)copper(II) (KOKGIB; Sarma & Baruah, 2008 ▸) and bis­(di­methyl­ammonium) bis­(μ_2_-3,5-di­carboxyl­atopyrazolato)dicopper(II) (ALERIU; Demir *et al.*, 2016 ▸).

## 6. Synthesis and crystallization

5-Methyl-3-pyrazolamine (0.015 g, 1.54 × 10 ^−4^ mol), copper(II) acetate (0.28 g, 1.54 × 10 ^−4^ mol) and di­ethano­lamine (0.032 g, 3.08 × 10 ^−4^ mol) were mixed together, and dissolved in water. After 3 days, clear, light-violet crystals were collected by filtration, dried for less than a minute, and then placed under crystallographic oil for further measurements.

## 7. Refinement

Crystal data, data collection and structure refinement details are summarized in Table 3 ▸. The H atoms of N_(p, c)_—H, C_p_—H and O_w_—H groups (p = pyrazole, c = carbamide, w = water) were positioned geometrically and refined as riding atoms, with C—H = 0.95 Å and *U*_iso_(H) = 1.2*U*_eq_(C) for C_p_—H groups, N—H = 0.88 Å and *U*_iso_(H) = 1.2*U*_eq_(N) for N_(p, c)_—H groups and O—H = 0.87 Å and *U*_iso_(H) = 1.5*U*_eq_(O) for O_w_—H groups. Methyl H atoms were positioned geometrically and were allowed to ride on C atoms and rotate around the C—C bond, with C—H = 0.98 Å and *U*_iso_(H) = 1.5*U*_eq_(C) for the CH_3_ groups.

## Supplementary Material

Crystal structure: contains datablock(s) I. DOI: 10.1107/S2056989023008575/tx2076sup1.cif

Structure factors: contains datablock(s) I. DOI: 10.1107/S2056989023008575/tx2076Isup2.hkl

CCDC reference: 2298123

Additional supporting information:  crystallographic information; 3D view; checkCIF report

## Figures and Tables

**Table 1:** Selected bond lengths and bond angles (Å, °)

Cu1—O1	1.9140 (17)	Cu1—N1	1.931 (2)
N1^i^—Cu1—N1	180.0	O1—Cu1—N1^i^	91.08 (8)
O1—Cu1—N1	88.92 (8)	N2—N1—Cu1	126.70 (16)

Symmetry codes: (i) −*x*, −*y*, −*z*

**Table 2:** Hydrogen-bond geometry (Å, °)

*D*—H⋯*A*	*D*—H	H⋯*A*	*D*⋯*A*	*D*—H⋯*A*
O4—H4*D*⋯O3^i^	0.87	1.80	2.664 (3)	171
O4—H4*E*⋯O1^ii^	0.87	2.44	2.930 (3)	116
O4—H4*E*⋯O2^ii^	0.87	2.02	2.873 (3)	167
N2—H2⋯O4^iii^	0.88	1.99	2.863 (3)	169
N3—H3⋯O2^iv^	0.88	2.02	2.889 (3)	169
O3—H3*A*⋯O2	0.87	1.89	2.756 (3)	176
O3—H3*B*⋯O4	0.87	1.92	2.783 (3)	169
C2—H2*A*⋯O3^iv^	0.95	2.43	3.340 (4)	159

Symmetry codes: (i) 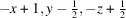 ; (ii) 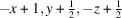 ; (iii) 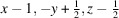 ; (iv) 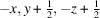 .

**Table 3:** Experimental details

Crystal data	
Chemical formula	[Cu(C_5_H_6_N_3_O_2_)_2_]·4H_2_O
*M*_r_	415.86
Crystal system, space group	Monoclinic, *P*2_1_/*c*
Temperature (K)	200
*a*, *b*, *c* (Å)	8.4623 (2), 5.64870 (16), 17.4536 (4)
β (°)	98.786 (2)
*V* (Å^3^)	824.51 (4)
*Z*	2
Radiation type	Cu *K*α
μ (mm^−1^)	2.39
Crystal size (mm)	0.15 × 0.15 × 0.15

Data collection	
Diffractometer	XtaLAB Synergy, Dualflex, HyPix
Absorption correction	Multi-scan (*CrysAlis PRO*; Rigaku OD, 2023 ▸)
*T*_min_, *T*_max_	0.638, 1.000
No. of measured, independent and observed [*I* > 2σ(*I*)] reflections	5223, 1634, 1401
*R*_int_	0.042
(sin θ/λ)_max_ (Å^−1^)	0.631

Refinement	
*R*[*F* ^2^ > 2σ(*F* ^2^)], *wR*(*F* ^2^), *S*	0.041, 0.118, 1.05
No. of reflections	1634
No. of parameters	116
No. of restraints	2
H-atom treatment	H-atom parameters constrained
Δρ_max_, Δρ_min_ (e Å^−3^)	0.45, −0.57

Computer programs: *CrysAlis PRO* (Rigaku OD, 2023 ▸), *SHELXT2018/2* (Sheldrick, 2015*b* ▸), *SHELXL2018/3* (Sheldrick, 2015*a* ▸) and *OLEX2* (Dolomanov *et al.*, 2009 ▸).

## supplementary crystallographic information

### Crystal data

**Table:**

[Cu(C_5_H_6_N_3_O_2_)_2_]·4H_2_O	*F*(000) = 430
*M**_r_* = 415.86	*D*_x_ = 1.675 Mg m^−^^3^
Monoclinic, *P*2_1_/*c*	Cu *K*α radiation, λ = 1.54184 Å
*a* = 8.4623 (2) Å	Cell parameters from 2285 reflections
*b* = 5.64870 (16) Å	θ = 5.1–72.3°
*c* = 17.4536 (4) Å	µ = 2.39 mm^−^^1^
β = 98.786 (2)°	*T* = 200 K
*V* = 824.51 (4) Å^3^	Block, clear light violet
*Z* = 2	0.15 × 0.15 × 0.15 mm

### Data collection

**Table:**

XtaLAB Synergy, Dualflex, HyPix diffractometer	1401 reflections with *I* > 2σ(*I*)
Detector resolution: 10.0000 pixels mm^-1^	*R*_int_ = 0.042
ω scans	θ_max_ = 76.8°, θ_min_ = 5.1°
Absorption correction: multi-scan (CrysAlisPro; Rigaku OD, 2023)	*h* = −10→10
*T*_min_ = 0.638, *T*_max_ = 1.000	*k* = −6→4
5223 measured reflections	*l* = −16→22
1634 independent reflections	

### Refinement

**Table:**

Refinement on *F*^2^	Primary atom site location: dual
Least-squares matrix: full	Hydrogen site location: mixed
*R*[*F*^2^ > 2σ(*F*^2^)] = 0.041	H-atom parameters constrained
*wR*(*F*^2^) = 0.118	*w* = 1/[σ^2^(*F*_o_^2^) + (0.0636*P*)^2^ + 0.4306*P*] where *P* = (*F*_o_^2^ + 2*F*_c_^2^)/3
*S* = 1.05	(Δ/σ)_max_ < 0.001
1634 reflections	Δρ_max_ = 0.45 e Å^−^^3^
116 parameters	Δρ_min_ = −0.57 e Å^−^^3^
2 restraints	

### Special details

**Table:**

Geometry. All esds (except the esd in the dihedral angle between two l.s. planes) are estimated using the full covariance matrix. The cell esds are taken into account individually in the estimation of esds in distances, angles and torsion angles; correlations between esds in cell parameters are only used when they are defined by crystal symmetry. An approximate (isotropic) treatment of cell esds is used for estimating esds involving l.s. planes.

### Fractional atomic coordinates and isotropic or equivalent isotropic displacement parameters (Å^2^)

**Table:**

	*x*	*y*	*z*	*U*_iso_*/*U*_eq_	
Cu1	0.000000	0.000000	0.000000	0.0327 (2)	
O4	0.6601 (3)	0.2829 (4)	0.29517 (11)	0.0518 (6)	
H4D	0.663197	0.160498	0.265176	0.078*	
H4E	0.733747	0.377068	0.282986	0.078*	
N2	−0.2338 (3)	0.3798 (4)	−0.05018 (12)	0.0354 (5)	
H2	−0.253149	0.326001	−0.098018	0.042*	
N1	−0.1348 (2)	0.2716 (4)	0.00870 (11)	0.0331 (5)	
N3	−0.0507 (3)	0.3550 (4)	0.14235 (11)	0.0362 (5)	
H3	−0.059104	0.455034	0.180169	0.043*	
O3	0.3513 (3)	0.4313 (5)	0.30719 (12)	0.0567 (6)	
H3A	0.280457	0.334940	0.282998	0.085*	
H3B	0.442727	0.365820	0.303128	0.085*	
C1	−0.1376 (3)	0.4090 (5)	0.07052 (14)	0.0327 (5)	
C2	−0.2365 (3)	0.6047 (5)	0.05156 (15)	0.0368 (6)	
H2A	−0.257394	0.729896	0.084961	0.044*	
C4	−0.4112 (3)	0.7265 (6)	−0.07931 (17)	0.0455 (7)	
H4A	−0.388986	0.707721	−0.132463	0.068*	
H4B	−0.398069	0.893005	−0.063909	0.068*	
H4C	−0.521048	0.676241	−0.076675	0.068*	
C3	−0.2977 (3)	0.5781 (5)	−0.02590 (15)	0.0360 (6)	
C5	0.0462 (3)	0.1642 (5)	0.16088 (13)	0.0339 (6)	
O1	0.0677 (2)	0.0130 (3)	0.10968 (10)	0.0391 (5)	
O2	0.1152 (2)	0.1456 (4)	0.22973 (9)	0.0400 (5)	

### Atomic displacement parameters (Å^2^)

**Table:**

	*U*^11^	*U*^22^	*U*^33^	*U*^12^	*U*^13^	*U*^23^
Cu1	0.0378 (3)	0.0405 (4)	0.0182 (3)	0.0035 (2)	−0.0012 (2)	−0.0008 (2)
O4	0.0577 (12)	0.0586 (14)	0.0355 (10)	−0.0059 (11)	−0.0042 (9)	−0.0031 (9)
N2	0.0403 (11)	0.0409 (13)	0.0228 (10)	0.0015 (10)	−0.0020 (8)	0.0030 (9)
N1	0.0363 (11)	0.0417 (12)	0.0200 (9)	0.0032 (9)	−0.0003 (8)	0.0016 (9)
N3	0.0442 (12)	0.0438 (13)	0.0196 (9)	0.0015 (10)	0.0016 (8)	−0.0031 (9)
O3	0.0618 (14)	0.0628 (14)	0.0418 (12)	−0.0098 (12)	−0.0036 (10)	0.0008 (11)
C1	0.0341 (12)	0.0392 (14)	0.0245 (11)	−0.0040 (11)	0.0036 (9)	−0.0001 (11)
C2	0.0393 (13)	0.0412 (15)	0.0296 (12)	0.0004 (12)	0.0046 (10)	−0.0009 (11)
C4	0.0419 (14)	0.0491 (18)	0.0435 (15)	0.0032 (13)	−0.0004 (12)	0.0085 (13)
C3	0.0352 (12)	0.0391 (14)	0.0336 (13)	−0.0027 (11)	0.0048 (10)	0.0041 (12)
C5	0.0409 (13)	0.0417 (14)	0.0187 (11)	−0.0064 (12)	0.0031 (9)	0.0000 (10)
O1	0.0478 (11)	0.0465 (12)	0.0205 (9)	0.0065 (8)	−0.0028 (8)	−0.0009 (7)
O2	0.0491 (10)	0.0501 (11)	0.0183 (8)	−0.0039 (9)	−0.0026 (7)	0.0022 (8)

### Geometric parameters (Å, º)

**Table:**

Cu1—N1	1.931 (2)	N3—C5	1.363 (4)
Cu1—N1^i^	1.931 (2)	O3—H3A	0.8697
Cu1—O1	1.9140 (17)	O3—H3B	0.8700
Cu1—O1^i^	1.9140 (17)	C1—C2	1.396 (4)
O4—H4D	0.8701	C2—H2A	0.9500
O4—H4E	0.8698	C2—C3	1.380 (4)
N2—H2	0.8800	C4—H4A	0.9800
N2—N1	1.367 (3)	C4—H4B	0.9800
N2—C3	1.341 (4)	C4—H4C	0.9800
N1—C1	1.333 (3)	C4—C3	1.490 (4)
N3—H3	0.8800	C5—O1	1.269 (3)
N3—C1	1.387 (3)	C5—O2	1.258 (3)
			
N1^i^—Cu1—N1	180.0	N1—C1—C2	110.7 (2)
O1^i^—Cu1—N1^i^	88.92 (8)	N3—C1—C2	127.2 (2)
O1^i^—Cu1—N1	91.08 (8)	C1—C2—H2A	127.2
O1—Cu1—N1	88.92 (8)	C3—C2—C1	105.5 (2)
O1—Cu1—N1^i^	91.08 (8)	C3—C2—H2A	127.2
O1—Cu1—O1^i^	180.0	H4A—C4—H4B	109.5
H4D—O4—H4E	104.5	H4A—C4—H4C	109.5
N1—N2—H2	124.3	H4B—C4—H4C	109.5
C3—N2—H2	124.3	C3—C4—H4A	109.5
C3—N2—N1	111.5 (2)	C3—C4—H4B	109.5
N2—N1—Cu1	126.70 (16)	C3—C4—H4C	109.5
C1—N1—Cu1	127.60 (17)	N2—C3—C2	107.0 (2)
C1—N1—N2	105.3 (2)	N2—C3—C4	121.7 (2)
C1—N3—H3	116.4	C2—C3—C4	131.4 (3)
C5—N3—H3	116.4	O1—C5—N3	120.7 (2)
C5—N3—C1	127.2 (2)	O2—C5—N3	117.9 (2)
H3A—O3—H3B	104.5	O2—C5—O1	121.4 (3)
N1—C1—N3	122.1 (2)	C5—O1—Cu1	132.59 (17)
			
Cu1—N1—C1—N3	−7.3 (4)	C1—N3—C5—O1	1.3 (4)
Cu1—N1—C1—C2	172.53 (18)	C1—N3—C5—O2	−179.9 (2)
N2—N1—C1—N3	179.7 (2)	C1—C2—C3—N2	−1.3 (3)
N2—N1—C1—C2	−0.4 (3)	C1—C2—C3—C4	179.3 (3)
N1—N2—C3—C2	1.1 (3)	C3—N2—N1—Cu1	−173.46 (18)
N1—N2—C3—C4	−179.4 (2)	C3—N2—N1—C1	−0.5 (3)
N1—C1—C2—C3	1.1 (3)	C5—N3—C1—N1	0.0 (4)
N3—C1—C2—C3	−179.1 (3)	C5—N3—C1—C2	−179.9 (3)
N3—C5—O1—Cu1	5.3 (4)	O2—C5—O1—Cu1	−173.44 (18)

Symmetry code: (i) −*x*, −*y*, −*z*.

### Hydrogen-bond geometry (Å, º)

**Table:**

*D*—H···*A*	*D*—H	H···*A*	*D*···*A*	*D*—H···*A*
O4—H4*D*···O3^ii^	0.87	1.80	2.664 (3)	171
O4—H4*E*···O1^iii^	0.87	2.44	2.930 (3)	116
O4—H4*E*···O2^iii^	0.87	2.02	2.873 (3)	167
N2—H2···O4^iv^	0.88	1.99	2.863 (3)	169
N3—H3···O2^v^	0.88	2.02	2.889 (3)	169
O3—H3*A*···O2	0.87	1.89	2.756 (3)	176
O3—H3*B*···O4	0.87	1.92	2.783 (3)	169
C2—H2*A*···O3^v^	0.95	2.43	3.340 (4)	159

Symmetry codes: (ii) −*x*+1, *y*−1/2, −*z*+1/2; (iii) −*x*+1, *y*+1/2, −*z*+1/2; (iv) *x*−1, −*y*+1/2, *z*−1/2; (v) −*x*, *y*+1/2, −*z*+1/2.

## References

[bb1] Aghaie, M., Rezaei, N. & Zendehboudi, S. (2018). *Renew. Sustain. Energy Rev.* **96**, 502–525.

[bb2] Cannone, S. F., Lanzini, A. & Santarelli, M. (2021). *Energies* **14**, 387.

[bb3] Conway, W., Fernandes, D., Beyad, Y., Burns, R., Lawrance, G., Puxty, G. & Maeder, M. (2013). *J. Phys. Chem. A*, **117**, 806–813. 10.1021/jp310560b 23286883

[bb4] Conway, W., Wang, X., Fernandes, D., Burns, R., Lawrance, G., Puxty, G. & Maeder, M. (2011). *J. Phys. Chem. A*, **115**, 14340–14349. 10.1021/jp2081462 22035132

[bb5] Dell’Amico, D. B., Calderazzo, F., Labella, L., Marchetti, F. & Pampaloni, G. (2003). *Chem. Rev.* **103**, 3857–3898. 10.1021/cr940266m 14531715

[bb6] Demir, S., Çepni, H. M., Hołyńska, M. & Kavanoz, M. (2016). *Z. Naturforsch.* **71**, 305–310.

[bb7] Dolomanov, O. V., Bourhis, L. J., Gildea, R. J., Howard, J. A. K. & Puschmann, H. (2009). *J. Appl. Cryst.* **42**, 339–341.

[bb8] Groom, C. R., Bruno, I. J., Lightfoot, M. P. & Ward, S. C. (2016). *Acta Cryst.* B**72**, 171–179. 10.1107/S2052520616003954 PMC4822653 27048719

[bb9] Kronberger, B., Johansson, E., Löffler, G., Mattisson, T., Lyngfelt, A. & Hofbauer, H. (2004). *Chem. Eng. Technol.* **27**, 1318–1326.

[bb10] Mani, F., Peruzzini, M. & Stoppioni, P. (2006). *Green Chem.* **8**, 995–1000.

[bb11] McCann, N., Phan, D., Wang, X., Conway, W., Burns, R., Attalla, M., Puxty, G. & Maeder, M. (2009). *J. Phys. Chem. A*, **113**, 5022–5029. 10.1021/jp810564z 19338322

[bb12] Mikkelsen, M., Jørgensen, M. & Krebs, F. C. (2010). *Energy Environ. Sci.* **3**, 43–81.

[bb13] Milner, P. J., Siegelman, R. L., Forse, A. C., Gonzalez, M. I., Runčevski, T., Martell, J. D., Reimer, J. A. & Long, J. R. (2017). *J. Am. Chem. Soc.* **139**, 13541–13553. 10.1021/jacs.7b07612 PMC8221660 28906108

[bb14] Ochedi, F. O., Yu, J., Yu, H., Liu, Y. & Hussain, A. (2021). *Environ. Chem. Lett.* **19**, 77–109.

[bb15] Qin, G.-F., Qin, Q.-Y., Long, B.-F., Wei, D.-P., Xu, Y.-H., Bao, S.-J. & Yin, X.-H. (2017). *J. Iran. Chem. Soc.* **14**, 1227–1234.

[bb26] Rigaku OD (2023). *CrysAlis PRO*. Rigaku Oxford Diffraction, Yarnton, England.

[bb16] Sarma, R. & Baruah, J. B. (2008). *J. Coord. Chem.* **61**, 3329–3335.

[bb17] Sheldrick, G. M. (2015*a*). *Acta Cryst.* A**71**, 3–8.

[bb18] Sheldrick, G. M. (2015*b*). *Acta Cryst.* C**71**, 3–8.

[bb19] Song, C., Liu, Q., Deng, S., Li, H. & Kitamura, Y. (2019). *Renew. Sustain. Energy Rev.* **101**, 265–278.

[bb20] Spackman, P. R., Turner, M. J., McKinnon, J. J., Wolff, S. K., Grimwood, D. J., Jayatilaka, D. & Spackman, M. A. (2021). *J. Appl. Cryst.* **54**, 1006–1011. 10.1107/S1600576721002910 PMC8202033 34188619

[bb21] Sreedhar, I., Vaidhiswaran, R., Kamani, B. M. & Venugopal, A. (2017). *Renew. Sustain. Energy Rev.* **68**, 659–684.

[bb22] Tang, Y., Kassel, W. S., Zakharov, L. N., Rheingold, A. L. & Kemp, R. A. (2005). *Inorg. Chem.* **44**, 359–364. 10.1021/ic048830r 15651882

[bb23] Wang, Y., Zhao, L., Otto, A., Robinius, M. & Stolten, D. (2017). *Energy Procedia*, **114**, 650–665.

[bb24] Zhang, R., Yang, Q., Liang, Z., Puxty, G., Mulder, R. J., Cosgriff, J. E., Yu, H., Yang, X. & Xue, Y. (2017). *Energy Fuels*, **31**, 11099–11108.

[bb25] Zhou, J.-H., Liu, Z., Li, Y.-Z., Song, Y., Chen, X.-T. & You, X.-Z. (2006). *J. Coord. Chem.* **59**, 147–156.

